# Home Bodies and Wanderers: Sympatric Lineages of the Deep-Sea Black Coral *Leiopathes glaberrima*

**DOI:** 10.1371/journal.pone.0138989

**Published:** 2015-10-21

**Authors:** Dannise V. Ruiz-Ramos, Miles Saunders, Charles R. Fisher, Iliana B. Baums

**Affiliations:** Biology Department, The Pennsylvania State University, University Park, Pennsylvania, United States of America; University of Texas, UNITED STATES

## Abstract

Colonial corals occur in a wide range of marine benthic habitats from the shallows to the deep ocean, often defining the structure of their local community. The black coral *Leiopathes glaberrima* is a long-lived foundation species occurring on carbonate outcrops in the Northern Gulf of Mexico (GoM). Multiple color morphs of *L*. *glaberrima* grow sympatrically in the region. Morphological, mitochondrial and nuclear ribosomal markers supported the hypothesis that color morphs constituted a single biological species and that colonies, regardless of color, were somewhat genetically differentiated east and west of the Mississippi Canyon. Ten microsatellite loci were used to determine finer-scale population genetic structure and reproductive characteristics. Gene flow was disrupted between and within two nearby (distance = 36.4 km) hardground sites and two sympatric microsatellite lineages, which might constitute cryptic species, were recovered. Lineage one was outbred and found in all sampled locations (N = 5) across 765.6 km in the Northern Gulf of Mexico. Lineage two was inbred, reproducing predominantly by fragmentation, and restricted to sites around Viosca Knoll. In these sites the lineages and the color phenotypes occurred in different microhabitats, and models of maximum entropy suggested that depth and slope influence the distribution of the color phenotypes within the Vioska Knolls. We conclude that *L*. *glaberrima* is phenotypically plastic with a mixed reproductive strategy in the Northern GoM. Such strategy might enable this long-lived species to balance local recruitment with occasional long-distance dispersal to colonize new sites in an environment where habitat is limited.

## Introduction

Characterizing biodiversity is important to understand ecosystem function and species interactions; but biodiversity can be underestimated due to the presence of cryptic species, which are difficult to identify based on their morphological characters [1]. Sometimes, a cryptic species group may harbor slight genetic differences whose functional significance can only be understood when the members occur in sympatry [2]. Cryptic species are common in the marine environment partly due to the complications of *in situ* observation [2]. The difficulties in observing behavior and the degradation of morphological traits during preservation also complicate the description of marine species [1, 2], and these obstacles are even more prevalent for deep-sea taxa [3, 4].

Corals are one group in which cryptic diversity is common, mostly owing to the occurrence of variable growth forms, life histories and habitat use [2, 5–7]. The application of genetic markers for species delimitation has greatly contributed to the reassignment of species relationships that were not apparent from morphology alone [8–11]. For example, high morphological variation has complicated species characterization in *Porites*, *Pocillopora* and *Seriatopora* [6, 8, 11]. In these groups, distinct morphologies may be genetically indistinguishable, while similar morphologies can belong to different species [8, 11, 12]. Due to this morphological convergence, important ecological and functional differences went unnoticed in *Porites* species of the Eastern Pacific [6], and in *Seriatopora hystrix* in the Great Barrier Reef [11].

In the black corals, the Antipatharia, including the genus *Leiopathes*, differentiation at the species level is difficult because of morphological simplicity and high plasticity [13]. For example, size, shape and arrangement of skeletal spines are major taxonomical characters for species identification in the Antipatharia [14], but spines are minimal or absent in *Leiopathes* species [15]. In the Gulf of the Mexico (GoM), the presence of different color morphs and variation in branching pattern and polyp size suggest the possibility of undescribed species of *Leiopathes* [16].

The existence of cryptic species [2] further complicates estimates of population connectivity in the deep sea [3]. Some authors suggest that cosmopolitan species are common in the deep sea perhaps because low temperatures and high pressures increase the longevity of the planktonic larvae allowing for greater dispersal distances [17]. However, many taxa show significant population structure, as topography, depth, local currents and differences in oxygen concentrations can act as barriers to dispersal [17–19]. Although some deep-sea species have high dispersion rates across oceanic regions [20–22], some apparent cosmopolitan species are in fact genetically distinct species with similar morphology [4, 23, 24].

Previous connectivity studies of benthic organisms suggested panmixia among sites within the Northern Gulf of Mexico [20, 21, 25]. However, timing of reproduction, larval duration, seasonal circulation patterns and availability of substrate influence population subdivision [26–29], so it is difficult to predict, *a priori*, the extent to which populations of a given species are likely to be connected. Recent studies on octocoral biodiversity in the deep GoM found that octocoral species are segregated by depth and habitat [29–31]. Bathymetric patterns are common drivers of genetic differentiation in deep-sea and shallow-water corals, probably due to marked differences in temperature, sedimentation and food availability [2, 7, 30, 32, 33].

To assess the presence of previously unrecognized species in *Leiopathes* collections, we used morphological, mitochondrial and nuclear ribosomal markers to test the hypothesis that color morphs of *Leiopathes glaberrima* constitute one biological species. We then developed ten microsatellite loci for *L*. *glaberrima* to infer population genetic structure and reproductive characteristics. We recovered two distinct genetic lineages in sympatry and used geospatial analysis to understand the influence of environmental characteristics (depth, slope and aspect) on the distribution of genetic lineages and color phenotypes within sites.

## Methods

### Sample collection

**Fig 1:**
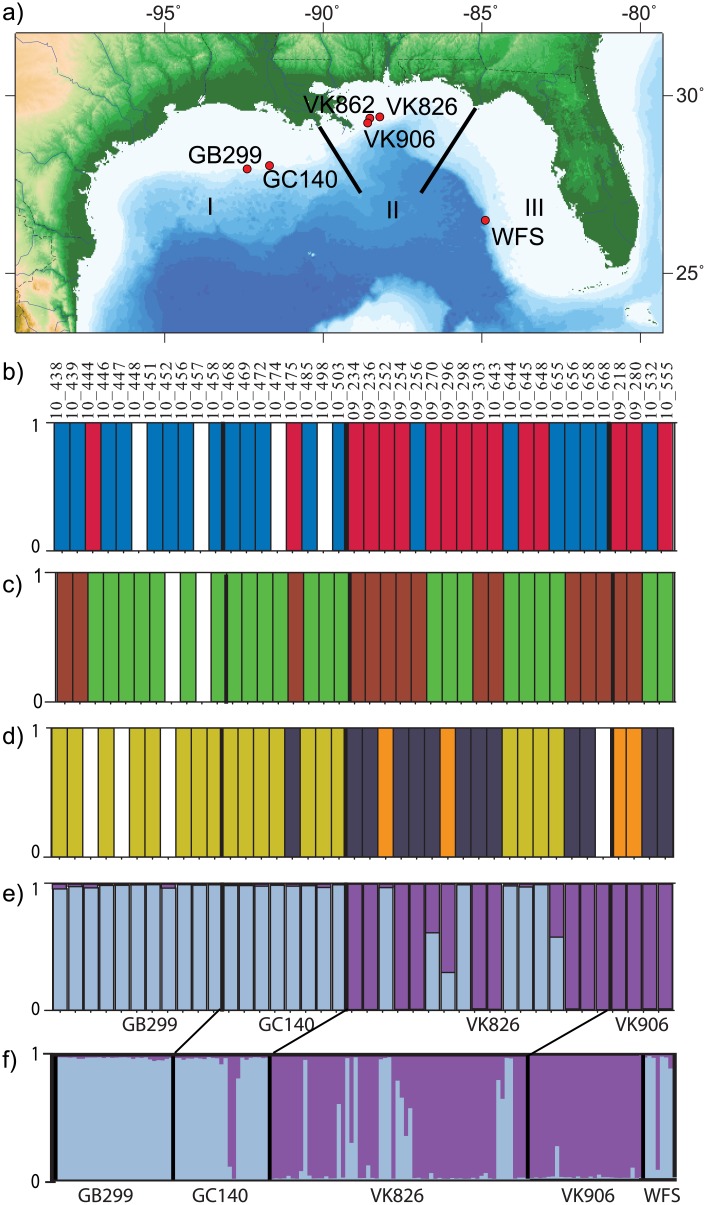
Collection sites in the Gulf of Mexico and sample clustering. (a) Sampling sites for *Leiopathes glaberrima* (inset) in the Gulf of Mexico (GoM). GB = Garden Banks, GC = Green Canyon, VK = Viosca Knoll, WFS = West Florida Slope. GB and GC are in biogeographic region I, VK is in biogeographic region II and WFS is within biogeographic region III [34]. (b) Color of the colonies (N = 40; red = red colonies, blue = white colonies) analyzed in (c)–(e), which included samples from biogeographic regions I and II. Genetic lineages identified by c) mitochondrial locus TRP (green = TRP lineage 1 and brown = TRP lineage 2) and (d) nuclear locus ITS-1 (grey = ITS-1 lineage 1, orange = ITS-1 lineage 2a, dark yellow = ITS-1 lineage 2b). White bars = sample not analyzed. (e) Probability of membership in K = 2 clusters of microsatellite genotypes recovered from the 40 *L*. *glaberrima* samples analyzed in (b)–(d). (f) Cluster analysis as in (e) of the full dataset of 148 *L*. *glaberrima* microsatellite genotypes from the GoM, WFS samples included in this analysis (N = 6). VK826 harbors colonies with ancestry in both lineages (M). VK862 excluded (N = 2). Light blue = microsatellite lineage 1, purple = microsatellite lineage 2.

Two hundred twenty two samples representing different color morphotypes of *Leiopathes glaberrima* were collected by the remotely operated vehicle (ROV) *Jason II* from the R/V Ron Brown (August 6 to September 12, 2009 and October 15 to November 1, 2010), and by the human occupied vehicle *Johnson Sea Link* from the R/V Seward Johnson (September 16 to 23, 2009 and September 22 to October 2, 2010). Permitting processes are not established for work on deep-sea corals in the Gulf of Mexico. Letters of acknowledgment were obtained from the National Oceanic and Atmospheric Administration for the research expeditions in accordance with the Magnuson-Stevens Fishery Conservation and Management Act. Samples of various colors were collected from depths between 248 m and 674 m, from the West Florida Slope (WFS) and locations named after the Bureau of Ocean Energy Management lease blocks in which they occurred: Garden Banks 299 (GB299), Green Canyon 140 (GC140), Viosca Knoll 826 (VK826), Viosca Knoll 906 (VK906) (Fig 1a and Table 1). Images of the colonies were taken during collection and shipboard. Tissue was preserved in 70% ethanol and stored at −80°C. Depth and location of the sample collections were noted at the time of collection and confirmed from dive logs.

**Table 1:** *Leiopathes glaberrima* samples from the Gulf of Mexico. Two lineages (L1 and L2) were identified with microsatellite (Msat) loci (unique genotypes shown in parenthesis). The total number of samples (N) and number of distinct multilocus genotypes, N_g_, as identified by microsatellite genotyping is indicated. Samples were categorized by sampling depth (D = 440–600 m, M = 300–440 m, S = 200–300 m) and colony color when available.

		Total	Msat Lineage (n)			Depth (n)			Color (n)		
Sites	GPS	N	L1	L 2	L3	D	M	S	Red	White	Unknown
GB299	27.69°N, −92.23°W	27	27(25)				26		1	16	5
GC140	27.81°N, −91.54°W	23	20(20)	2(2)	1			23	2	12	8
VK826	29.16°N, −88.02°W	75	9(8)	58(40)	8(8)	75			49	24	2
VK862		3		2(2)	1		3		2		1
VK906	29.07°N, −88.38°W	73		73(27)			73		37	34	2
WFL Slope	26.18°N, −84.71°W	6	5(5)	1		3	3		3	1	2
N		207	61	136	10	78	105	23	94	87	20
N_g_		150	58	72	8						
N_g_/N		0.72	0.95	0.53							

**Fig 2:**
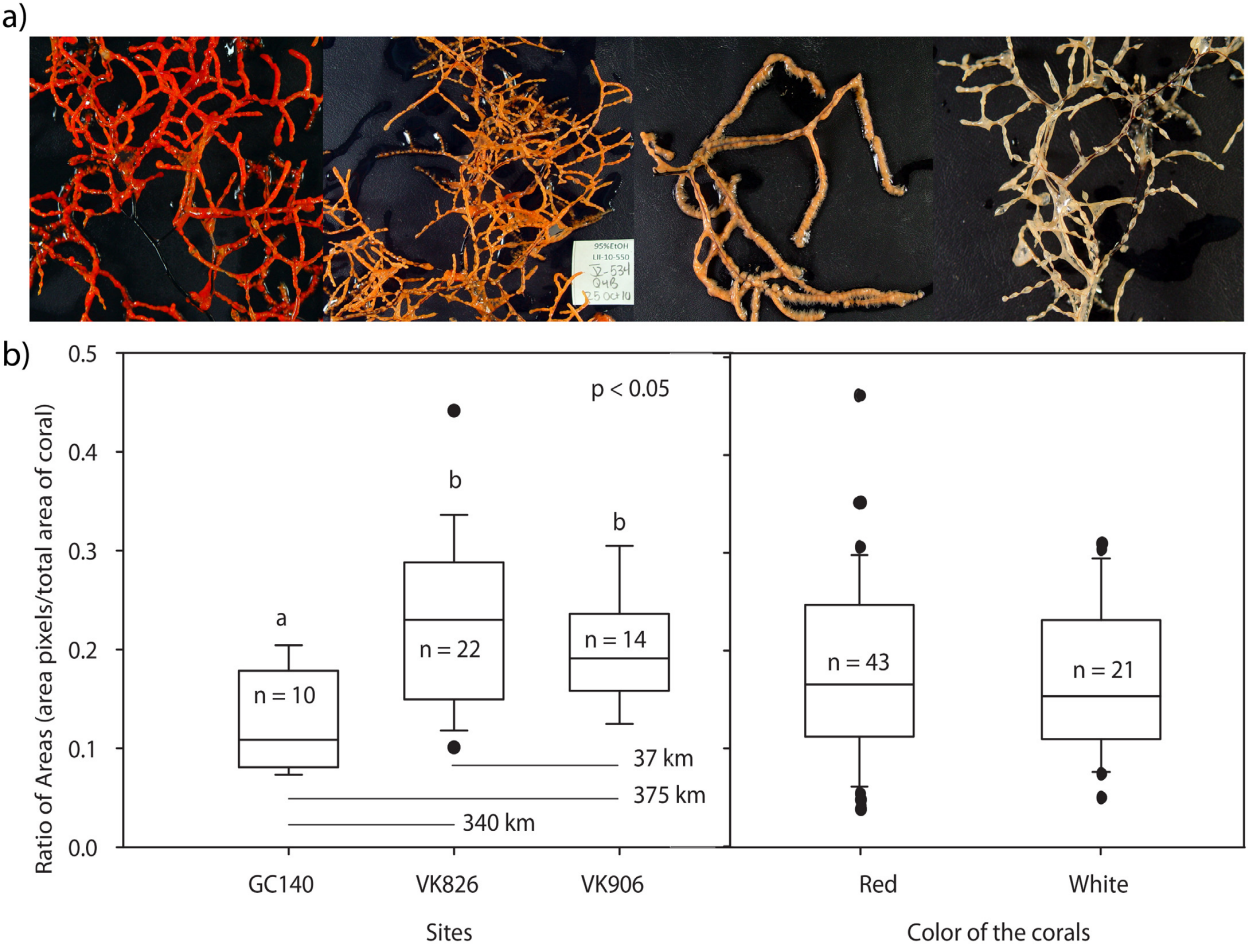
Variation in color and branch density for the morphotypes of *Leiopathes glaberrima*. a) Color morphotypes of *L*. *glaberrima*, from left to right: red, orange, light orange and white. b) Branch density of *L*. *glaberrima* from three sites was calculated from digitized photographs of colonies. The density of the branches was calculated by dividing the total area of pixels obtained and the square area occupied by the coral. Branch density was compared among sites with a one-way ANOVA. GC140 was different from the Viosca Knoll sites, 826 and 906. No difference in branching density was found between color morphotypes (see text).

Before collecting the colonies, they were identified as red or white from the ROV cameras. However, during collection we noticed that some white colonies were a pale yellow or light orange, while the red colonies varied from orange to dark red (Fig 2a). Wet lab photos were taken on a black background using a contrasting white and black scale and the white balance option, and the RGB (red, green and blue) contents analyzed using discriminant analysis. This analysis grouped all of the pale and light yellow-orange colonies together with the “white” colonies, and the orange to red colonies as a separate group. For subsequent analyses only these two groups, white and red, were compared.

### Species delimitation

Photographs of sampled colonies taken after collection on board the ships were analyzed for branching patterns. Branch density measurements were made on sixty-four samples from three sites (GC140, VK906 and VK826) and depths from 248 m to 548 m. The software Image J v. 1.43 [35] was used to measure the length and width of the sampled branches; the length and width were then multiplied to obtain the squared area outlined by the colony. Area was also measured with Axiovision software v. 4.8 (Carl Zeiss Microimaging, Inc.), after separating the pixels occupied by the coral branches from the background. The density of the branches was calculated by dividing the total area of pixels obtained with Axiovision and the square area occupied by the coral.

We preliminarily treated each color: red, orange, light yellow-orange, pale yellow and white as separate, and no difference in branch density was observed (one-way ANOVA, p = 0.12). To increase the power for detecting associations between morphology and color phenotype, we grouped colonies into the two RGB color groups: red (including red, and orange) and white (including white, pale yellow, and light yellow-orange colonies). We then reanalyzed the data, which are presented here. A t-test was used to compare the branch densities of color morphs (grouped as red or white), and a one-way ANOVA was used to compare the branch densities between depth and locations.

To determine species status of *L*. *glaberrima*, samples from two sites (VK826 and VK906) with diverse color morphs ranging from white to red were compared using three *Leiopathes*-specific mitochondrial markers: COI-COIII, ND5-ND2 and TRP (partial TrnW-ITS-NADH) [36, 37] and the coral-specific nuclear ribosomal ITS-1 marker [38]. See S1 Methods for amplification conditions. Amplification products were treated with Exo-Sap (GE Healthcare) and Sanger sequenced. Sequences were edited and aligned using Codon Code Aligner (CodonCode Corporation, Dedham, Massachusetts). Bayesian phylogenetic trees were constructed in MrBayes [39].

Data was coded as follows for statistical tests: color of the morphotypes (Red/White), TRP lineage (lineage 1/2), microsatellite lineage (lineage 1/2) and ITS1 lineage (lineage 1/2). Fisher’s exact tests were performed to test for associations between colors, geographical region [34] and genetic markers, followed by Bonferroni adjustment for multiple testing. Data were also analyzed as Generalized Linear Models using a binomial error distribution and logit link function in R [40] to test whether genetic lineage or geography predicts color. We fitted the complete model with the interaction terms first (Color ~ pop*msat*its*trp) and removed non-significant interactions by backward-elimination using Analysis of Variances (ANOVA) for generalized linear models in R. In cases of overdispersion, we used a quasibinomial distribution and logit link function.

### Population genetics

Ten polymorphic microsatellite loci (Genbank submission numbers KJ914618-KJ914627; S1 Table) were designed from sequence data obtained from genomic shotgun sequencing *of L*. *glaberrima* on a 454 GS-FLX sequencer. See S1 Methods for microsatellite design and amplification conditions. Fragments were analyzed using an ABI 3730 sequencer with an internal size standard (Genescan LIZ-500; Applied Biosystems). Genemapper 4.0 (Applied Biosystems) was used to visualize the electropherograms and score the alleles. Samples that failed to amplify 3 or more loci (N = 28, 13%) were excluded from the analyses.

Population genetic analyses were performed using the program GenAlEx v6.5 [41]. Exact matches at all loci were identified first and samples that shared the exact multilocus genotype (MLG) at all 10 loci were considered to be clonemates of the same genet. Using all 10 loci, GenAlEx estimated the probability of identity (or the probability that two samples share the same MLG even though they are not clones) as 10^−7^ across populations. Subsequent population genetic analyses were performed on unique MLGs only. Loci were tested for adherence to Hardy-Weinberg expectations and linkage disequilibrium (LD) with GenAlEx v6.5 and Genepop [42, 43], considering all unique MLGs and when analyzing microsatellite lineages 1 and 2 (see below) separately. When heterozygote deficits were observed, null allele frequencies and inbreeding coefficients were estimated using INEST [44]. RMES [45] was used to distinguish between inbreeding and self-fertilization.

Principal coordinate analysis (PCoA) was performed on a pairwise genetic distance matrix (F_st_) among all samples in GenAlEx v6.5. Population differentiation was estimated using Analysis of Molecular Variances (AMOVA) in GenAlEx v6.5. The number of genetically distinct clusters, K, were detected using the program Structure [46, 47]. The MCMC chain was run for 10^6^ steps and 10% of the chain was discarded as burn-in. Each K between 1 and 8 was repeated three times. Admixture was assumed and no location prior was specified, because the presence of multiple lineages per location was suspected. Structure results were analyzed in Structure Harvester [48] to find the most likely number of populations, K. The robustness of the Structure results was tested using two other clustering programs: Instruct, which models inbreeding and does not assume Hardy—Weinberg equilibrium [49] and Geneland [50, 51], which can account for null alleles but assumes uncorrelated allele frequencies [52]. For model configurations see captions of S1 Fig. Spatial clustering of groups and individuals were also performed in Baps [53].

To infer phylogenetic relationship using the microsatellite data we calculated the genotypic distances using the Bruvo distances method. Bruvo distances take into account mutation processes, and have been used to study species divergence and population structure [54, 55]. Neighbor-Joining Trees and Minimum Spanning Networks from the Bruvo distances were calculated using the R package Poppr [56].

Next, we created new datasets, each containing MLGs with > 0.8 probability of belonging to one of the two microsatellite lineages (L1: N = 61, L2: N = 76, unassigned N: 10), and ran the previously described population genetic analyses on each dataset using GenAlEx v6.5 and Genepop.

### Spatial analysis

*Leiopathes* survey transects were derived from frame grabs of down-looking video obtained using the ROV Jason II during 2009 and 2010 dives to the VK sites. The frame grabs and location data were obtained from the WHOI Jason VirtualVan online repository (http://4dgeo.whoi.edu/jason/). Survey points were manually selected at 10-meter intervals and time-matched to the frame grabs in the VirtualVan. Framegrabs were surveyed for the presence/absence, number and color of *L*. *glaberrima* colonies. The data was imported into ArcGIS Version 10.1 (ESRI 2011. ArcGIS Desktop: Release 10. Redlands, CA: Environmental Systems Research Institute) and overlaid on bathymetry (VK906 R/V Nancy Foster Multibeam was 5m pixel resolution, VK826 AUV Sentry Multibeam was 1m pixel resolution). The site bathymetry was processed through the Spatial Analyst extension to produce slope and aspect raster datasets that were imported into MaxEnt [57]. In addition to the three raster datasets, *L*. *glaberrima* collections made during the 2009 and 2010 Lophelia II Research Cruises were added to the MaxEnt program. The *L*. *glaberrima* collections dataset included the color, microsatellite lineage, clone id (as identified via microsatellite genotyping, see below), and the UTM coordinates for each collected colony. Each non-location attribute (color category, microsatellite lineage) was modeled with MaxEnt to identify the environmental characteristics associated with the occurrence of *L*. *glaberrima*. To test whether the model was significantly more robust than random (Area Under the Curve (AUC) > 0.5), a one-tailed one-sample t-test was performed.

## Results

### Species delimitation

Differences in branch densities were non-significant for the color categories (Mann-Whitney Rank Sum test, *p* = 0.75, N_white_ = 21, N_red_ = 43, *U* = 429). In contrast, branch densities were significantly different among sites (One-Way ANOVA, *p* < 0.004, N_GC140_ = 10, N_VK826_ = 22, N_VK906_ = 14, df_total_ = 45, *F* = 6.42), with GC140 differing from the two Viosca Knoll sites (*Post Hoc Tukey* HSD *test*, *p* < 0.05) (Fig 2b).

The mitochondrial markers COI-COIII (740 bp) and ND5-ND1 (660 bp) were monomorphic throughout the Gulf of Mexico (S2 Fig). The samples tested included red and white color morphs from the Viosca Knoll (VK) sites, VK826 and VK906. The third mtDNA locus, TRP (769 bp), recovered two mitotypes of *L*. *glaberrima* with four base pair differences (S2a Fig). The mitotypes (Fig 1c) were unrelated to color (white and red colonies were mixed within both mitotypes, Fig 1b; Fisher’s exact test, p = 0.18), but may be related to geographic regions (Factors: GB299/GC140 and VK sites, Fisher’s exact test p = 0.02).

In the ITS-1 nuclear region, we observed only seven polymorphic sites in a 782 bp alignment and resolved three lineages (S3 Fig). The recovered ITS-1 lineages may relate to color (Fisher’s exact test, p = 0.01) and geography (Fisher’s exact test, p < 0.001). After Bonferroni correction, color and geography remained significantly correlated.

Based on the Generalized Linear Models (GLM) color of the colonies was correlated with population of origin (*X* ^2^ = 4.22, p = 0.001) and ITS1 lineages (*X* ^2^ = 9.06, p = 0.01), but not mitochondrial lineage (*X* ^2^ = 0.25, p = 0.62).

### Population genetics

Microsatellite loci amplified reliably (N = 210, 0.05% overall failure rate, 13% of samples failed to amplify at more than 2 loci) and had, on average, 6.28 (+/- 0.53 SE) alleles per locus (S2 Table). Expected heterozygosity ranged from 0.26 to 0.90 (mean = 0.61 +/- 0.03 SE). The probability of identity ranged from 2.8×10^−3^ to 1×10^−9^ per population, indicating high power to distinguish between asexually produced individuals and those that were closely related (S2 Table). Null allele frequencies estimated on a dataset with unique MLGs ranged from 0.01 to 0.26 across loci (S4a Fig), and were similar across sites (one-way ANOVA, p = 0.22, WFS excluded). The frequency of null alleles was higher (> 0.20, INEST) at WFS, but this site had a limited number of MLGs (N = 6). Although null allele frequencies higher than 0.15 were observed at the sites with both lineages, these frequencies decreased when lineages were treated separately. Null allele frequency per MLG was low (F_is mean_ ≤ 0.01, 95% CI: 0.00–0.08, S4 Fig). Overall selfing rates were not different from zero (*s*_all_ = 0.07, 95% CI: 0.00–0.06) but when estimated within the individual populations, VK826 had a significant selfing rate (*s* = 0.11, 95% CI: 0.00–0.12, *p* = 0.02).

Structure (Fig 1e and 1f) and PCoA (S5 Fig) indicated the presence of two lineages (K = 2) in the dataset. Lineage 1 (L1) dominated GC140, GB299 and WFS, whereas VK906, was dominated by Lineage 2 (L2). VK826 harbored both lineages, and 10 samples showed evidence of hybridization (assignment probability of < 0.8). Instruct (which accounts for inbreeding), Geneland (account for null alleles) and Baps also suggested two lineages of *L*. *glaberrima* (S1 Fig).

The MSN facilitated the observation of the mutational steps between the lineages and corroborated the results from the assignment test. Although the lineages shared a few nodes, two main clusters were observed, corresponding to the two microsatellite lineages (S6 Fig). Analyses of molecular variances among sites suggested geographic differentiation was small, and significant only for L2 (Table 2), perhaps due to the now small sample sizes.

**Table 2:** Analysis of molecular variance (AMOVA) among sites and microsatellite lineages (L1 and L2). Within individual analysis was suppressed and significance evaluated based on 999 permutations across the whole dataset. % = percent of the estimated total variance.

	Source	DF	SS	MS	%	F_st_	F_st_ max	F'_st_
All	Among Pops	4	103.49	25.87	12	0.12**	0.38	0.32
	Within Pops	293	915.98	3.13	88			
	Total	297	1019.48		100			
L1	Among Pops	3	10.33	3.44	1	0.01	0.4	0.01
	Within Pops	118	352.11	2.98	99			
	Total	121	362.44		100			
L2	Among Pops	1	10.84	10.84	4	0.04**	0.41	0.09
	Within Pops	148	436	2.95	96			
	Total	149	446.84		100			

DF = degrees of freedom, SS = sum of squares, MS = mean sum of squares. Sites with < 5 genotypes excluded.

** *p* < 0.001.

**Fig 3:**
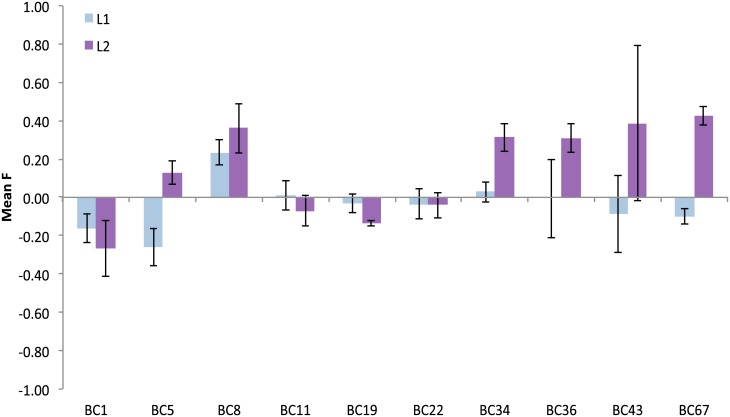
Mean Fixation indexes (F-values) for *Leiopathes glaberrima*. F-values were not significantly different from zero for most loci (BC1 –BC67) in L1. In L2 F-values consistently deviated from expectations. Given are means across loci and their standard errors. Fixation Index = (He - Ho) / He = 1 - (Ho/He) where He = expected heterozygosity and Ho = observed heterozygosity.

When analyzing all unique multilocus genotypes (MLGs), departures from Hardy-Weinberg equilibrium were significant in 23 out of 50 comparisons, mostly involving the two VK populations that harbored both microsatellite lineages. After separating the two lineages, only 2 out of 38 tests and were significant for L1 (Fig 3), while half of the 34 tests in L2 remained significant (Fig 3).

Null allele frequencies across the ten loci and lineages ranged from 0.01 to 0.26 (S4b Fig), and did not differ between L1 and L2 (Mann-Whitney Rank Sum test, N_L1_ = 10, N_L2_ = 10, U = 27.50, T = 82.50, *p* = 0.10). When testing loci individually, differences in null allele frequency were detected for BC67 (t = −3.47, df = 135, *p* < 0.001) and BC36 (t = −2.03, df = 135, *p* = 0.04). Null allele frequencies per MLG were low (F_is mean_ ≤ 0.01, 95% CI: 0.00 −0.07, S4 Fig), but significantly different between the lineages (Mann-Whitney Rank Sum Test, N_L1_ = 61, N_L2_ = 76, U = 1457.50, T = 3348.50, *p* < 0.001).

Estimates of selfing rates were not different from zero for L1 (*s* = 0.01, 95% CI: 0.00–0.06, *p* = 0.39). L2 showed moderate selfing rates (*s* = 0.11, 95% CI: 0.00–0.06, *p* = 0.003. The colonies with mixed ancestry (N = 10) also had moderate selfing rates (*s* = 0.18, 95% CI: 0.00–0.14, *p* = 0.05). Of the 210 samples genotyped, 20 had MLGs that occurred more than once. The ratio of the number of unique MLGs over the number of samples was 0.95 in L1 (N_g_ = 61, N = 64) and 0.57 in L2 (N_g_ = 78, N = 136). Note that sampling density was similar but not identical among sites (S3 Table) therefore no statistical comparisons of clonal structure were made among sites. Most of the potential clones were found in L2 at VK906, where the two largest genets had 15 and 21 members. In contrast, the largest number of clonemates per genet in L1 was 3 (GB299). Clones were only found within sites, and the largest distance between clonemates was 611 m in L2 (S3 Table).

### Spatial analysis of the Viosca Knoll sites

**Fig 4:**
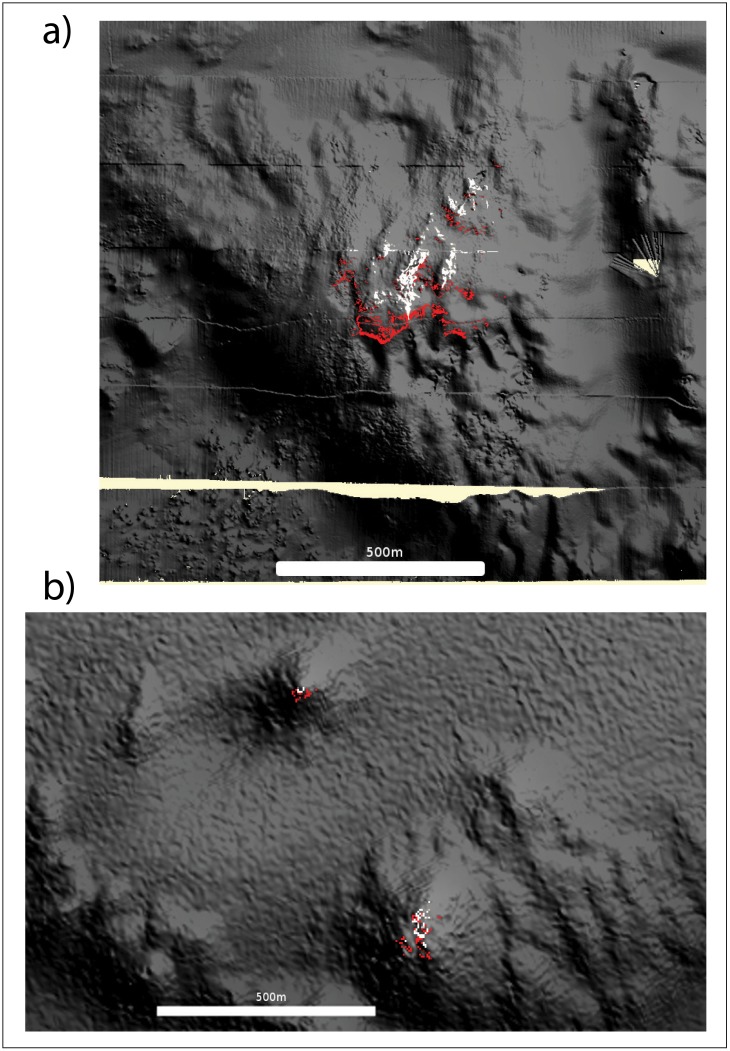
Maximum entropy model (MaxEnt) for the distribution of *Leiopathes glaberrima* within VK826 (a) and VK906 (b). Red pixels represent the 85% probability of occurrence for the red colonies, and white pixels the 85% probability of occurrence of the white colonies.

Red and white color morphs were distributed differently at the Viosca Knoll sites (Fig 4a and 4b). Red colonies dominated the southeast range of VK826, with white colonies restricted to the northern area of the site (Fig 4a). Results from the MaxEnt models suggested depth influenced distribution of white and red colonies at VK826 (Fig 4a). The distribution of red colonies was predicted by slope more so than the distribution of white colonies (22% versus 2%, Table 3). At VK906, the red colonies dominated on the slopes of the knoll, the southeast in particular; white colonies were concentrated at the top and eastern area of the knoll (Fig 4b). Accordingly, the MaxEnt models suggested depth also influenced distribution of white and red colonies at VK906 (Fig 4b). In addition, slope contributed 12% to the model prediction for the distribution of the red colonies but only 3% to the prediction for the distribution of the white colonies. Note that while relative depth influenced the distribution of color morphs at both VK sites, absolute depth differed between the sites (Table 1).

**Table 3:** Validation statistics and Jack-knife analysis of the variable contributions. Values for the regularized training gain of the Jack-knife test indicates the variable contribution to the model, the higher the value the greater the contribution (values are not directly comparable between the different sites). Standard deviations in parentheses.

Variable	VK826					VK906		
*Validation Statistics*	Color		Lineages			Color		Lineages
Test AUC	0.84* (0.07)		0.86* (0.07)			0.94* (0.04)		0.98* (0.01)
*Validation Statistics*	Red	White	L1	L2	L3	Red	White	L2
Test AUC	0.81* (0.06)	0.87 (0.03)	0.94* (0.03)	0.86 (0.03)	0.79 (0.13)	0.95* (0.03)	0.93* (0.05)	0.98* (8.2^−03^)
Test gain	0.54	0.84	1.63	0.85	-0.08	2.68	2.94	3.42
10th percentile training presence	25.80	31.80	20.60	22.77	18.32	21.42	18.48	19.20
Omission rate (Threshold 10)	9%	9%	0%	8%	0%	6%	10%	7%
*Percent contribution*	Red	White	L1	L2	L3	Red	White	L2
depth (1m utm16)	58.30	85.20	97.50	55.40	71.20	79.50	94.40	90.70
slope	24.70	2.10	1.40	26.40	27.80	11.50	0.50	4.90
aspect	17	12.60	1.10	18.20	0.90	9	5	4.40
*Jack-knife of variable importance*	Red	White	L1	L2	L3	Red	White	L2
depth (1m utm16)	0.49	0.68	1.44	0.61	0.46	2.20	2.59	3.20
slope	0.23	0.01	0.00	0.31	0.04	0.50	0.02	0.40
aspect	0.10	0.13	0.00	0.15	0.00	0.02	0.01	0.00
*Test AUC for a single variable*	Red	White	L1	L2	L3	Red	White	L2
depth (1m utm16)	0.78*	0.85*	0.94*	0.77*	0.82	0.92*	0.95*	0.97*
slope	0.63*	0.56*	0.44	0.71*	0.43	0.75	0.70	0.75*
aspect	0.58	0.64	0.46	0.60	0.64	0.67	0.54	0.62

*Values are significantly different from random prediction (One-Sample t-test, p < 0.01, One-Sample Signed Rank test, p < 0.03 if normality failed).

Lineage 1 was restricted to the northern area of VK826, and L2 was widespread over the slope of VK826 (Fig 4). Again, depth was identified as the variable influencing the distribution of both lineages at each site, but slope had a significant contribution to the distribution of L2 only (Table 3). Depth and slope also influenced the distribution of L2 at VK906 (Table 3).

## Discussion

The black coral, *Leiopathes glaberrima*, is an important foundation species in the deep Gulf of Mexico. Here, we discovered the presence of two microsatellite lineages growing in sympatry in the northern region of the Gulf. The assignment of sympatric individuals to two genetic lineages suggested barriers to gene flow, with lineage 2 restricted to the Viosca Knolls. Indications of limited mating between the two lineages came from the occurrence of individuals with mixed ancestry and the genotypic distance between samples. We propose two possible explanations for the segregation patterns: we might be observing incipient ecological divergence as a consequence of limited suitable habitat or a species with mixed mating strategy.

### Ecological divergence

Recent advances in genetic tools have led to the discovery of several cryptic lineages within morphologically similar collections of corals [6–8, 11]. In some of these corals, the lineages inhabit different habitats distinguished by differences in depth [7, 29, 58], wave action [11, 59], and latitude [6]. In some cases, as with the morphologically similar species *Porites lobata* and *Porites evermanni* in the Eastern Pacific, the species differ in the ecological niches they occupy and in the amount of asexual reproduction [6]. *P*. *evermanni*, the species with higher frequency of asexual reproduction is able to persist in marginal environments, perhaps because locally adapted genotypes do not require the presence of a sexual partner to proliferate and dominate local communities [6, 59]. Like in the previous examples, the two lineages of *L*. *glaberrima* might occupy different niches in the Gulf of Mexico.

L1 is the dominant lineage across the Northern Gulf of Mexico, except in the Viosca Knolls where L2 is dominant. In the Viosca Knoll region, L1 and the colonies with mixed ancestry (M) appear to be restricted to the northern edge of the VK826 site (Fig 4, at this site L1 colonies were mostly white), while L2 is found on the slopes of both VK826 and the VK906. The distribution of L1 across the Northern GoM, and its lack of population structure suggest the lineage is capable of long-distance dispersal, while self-fertilization in L2 would facilitate local recruitment in a new habitat. The combination of local and long-distance dispersal is a common strategy of long-lived species to maintain genetic diversity while colonizing new habitats [60].

In the Viosca Knolls, depth and slope influenced the distribution of the color morphotypes of *L*. *glaberrima*, but not the microsatellite lineages. In previous habitat distribution models, depth and temperature had high predictive power for the distribution of coral colonies [30, 61]. The relatively small difference in depth within the Viosca Knolls sites is unlikely to prevent gene flow, but the difference in local topography and associated ecological factors might be enough to influence the distribution of the phenotypically distinct colonies.

Cold water corals are often observed on and around topographic highs, as these features can increase current velocity and facilitate filter feeding [62]. Differences in feeding strategies dependent on the local microenvironment might explain the presence of multiple color morphs: color might not be host-generated but derived from ingested food or from microbial symbionts [63, 64]. Branching patterns are also influenced by the location of the colonies in relation to water currents and the amount of suspended food particles [5, 65, 66]. Here, phenotypic variation in relation to color and branch densities was observed among corals in different sites, which might result from variations in the local environment (in relation to food and flow). In the same manner, the distribution and recruitment of the lineages might be influenced by these local patterns of topography and current velocity.

### Mixed sexual reproductive strategy

Mixed-mating systems are often found in plants and fungi [67–71], where hermaphroditic species reproduce by both self- and cross-fertilization [72, 73]. Mixed-mating systems arise when the advantages of self-fertilization are greater than the disadvantages of inbreeding depression, which is usually the case for isolated populations [72, 74].

The Anthozoa (corals, black corals and anemones) includes gonochoric and hermaphroditic species with either internal or external fertilization [75, 76]. Polyps of gonochoric species have either male or female gonads, while polyps of simultaneous hermaphroditic species harbor gonads of both sexes. Some species, like *Diploastrea heliopora*, are unusual in that polyps may harbor male, female or hermaphroditic gonads [77]. This mode of mixed sexuality might promote outbreeding during times of high population densities, while allowing for self-fertilization during population declines [78].

As in plants, self-fertilization in corals can be autogamous (between gametes from the same polyp) and geitonogamous (between gametes from different polyps of the same colony) [75]. Most black corals colonies are gonochoric or sequential hermaphrodites [79], which are inherently incapable of autogamy. But in sequentially hermaphroditic corals, sex changes occur with changes in colony size, or in response to energetic or environmental constrains [80, 81]. If ramets of a genet have different sexes, then reproduction among ramets would result in geitonogamous self-fertilization [82].

Here, our MLG data show some evidence of a mixed mating strategy in *Leiopathes glaberrima*. The two microsatellite lineages of *L*. *glaberrima* differed in their adherence to Hardy-Weinberg equilibrium assumptions (Fig 3). Lineage 1 conformed to the expectations of an outbred species (once clones are removed from the dataset), while L2 was highly inbred. Members of L2 were more closely related to each other than members of L1 (S6 Fig). Recall that L2 was mostly restricted to VK906, a relatively small mound, about 100 m in diameter rising 30 m from the surrounding seafloor. The combination of limited habitat and relative isolation of this site might explain the increased inbreeding found in L2.

Differences in selfing rates and inbreeding coefficients between the two lineages could signify the presence of a mixed-mating system. To our knowledge, this is the first description of a coral species with sympatric, genetically distinguishable lineages that may utilize different reproductive strategies. Such observations have been made in plants. For example, in some populations of the coastal plant *Camissoniopsis cheiranthifolia* two distinct floral phenotypes with high and low selfing rates co-occur [83]. In other parts of the range, one or the other floral phenotype may be found, with their associated selfing rate [83]. Possible signs of self-fertilization in a black coral like *L*. *glaberrima* were unexpected. Until more detailed reproductive studies of *L*. *glaberrima* are performed, we cannot distinguish between self-fertilization within polyps or between clonemates, but clonal geitonogamy is likely to occur.

In summary, we discovered the presence of two sympatric lineages that differ in clone density, selfing rates, inbreeding coefficients, and might occupy different niches in the Gulf of Mexico.

## Supporting Information

S1 Fig Population genetic analysis of *L*. *glaberrima* genotypes using probability of membership clustering methods. (a) InStruct plot showing the probability of membership (y-axis) of each of the samples (x-axis) with inbreeding calculations, (K = 2). InStruct run in three parallel chains with 10^6^ MCMC repetitions and a burn-in of 10^5^ iterations each. (b) Spatial clustering of groups and individuals estimated in Baps under population mixture (b) and population admixture models (c), with a maximum of K = 7, (K = 2). (d) The optimal number of clusters estimated by Geneland without specifying any priors was 2 (K = 2).
(EPS)

S2 Fig Bayesian phylogenies of mitochondrial markers COI-COIII and ND5-ND1. (a) COI-COIII (740bp; GenBank Accession numbers: KF013048—KF013088) and (b) ND5-ND1 (660 bp, GenBank Accession numbers: KF012993—KF013047) showed very little sequence diversity in *L*. *glaberrima* samples from throughout the Gulf of Mexico resulting in star-shaped phylogenies. Sequences from *Antipathes griggi* were used as the out-group.
(EPS)

S3 Fig Bayesian phylogenies of mitochondrial TRP and nuclear ribosomal ITS-1. (a) Bayesian phylogeny of a portion of the mitochondrial TRP gene (trnW-nad2; 730 bp; GenBank Accession numbers: KF013089—KF013130) revealed two lineages within the sampled *L*. *glaberrima*, distinct by one amino acid change. Numbers represent posterior probabilities. The two lineages were unrelated to color of the colonies (indicated by the color of the labels, red = red colonies, blue = white colonies, black = color unknown), sampling site (VK906, VK826) or microsatellite lineage designation (M1 = microsatellite lineage 1, M2 = microsatellite lineage 2, M3 = mixed ancestry). (b) Bayesian phylogeny of a portion of the nuclear ribosomal ITS-1 gene (769 bp; GenBank Accession numbers: KJ650006—KJ650026) revealed three clusters within *L*. *glaberrima*, distinct by seven amino acid change and unrelated to color of the colonies (indicated by the color of the labels, red = red colonies, blue = white colonies, black = color unknown), sampling site (VK906, VK826) or microsatellite lineage designation (M1 = microsatellite lineage 1, M2 = microsatellite lineage 2, M3 = mixed ancestry). Numbers represent posterior probabilities. Sequences from *Chrysopathes formosa* (a) and *Antipathes griggi* (b) were used as the out-group.
(EPS)

S4 Fig Frequencies of null alleles per locus. (a) Frequency of inbreeding-adjusted null alleles per locus across the sampled sites in the Northern GoM, estimated on a dataset with unique multi-locus genotypes (Individual Inbreeding Model-based estimates after 100000 iterations). The differences in the mean values among the null allele frequency within loci across the sampled sites are not big enough to exclude the possibility that the difference is due to random sampling (One-way ANOVA, *p* = 0.22, F = 1.53, df = 3). (b) Frequency of null alleles per locus for the two microsatellite lineages of *L*. *glaberrima*, estimated on a dataset with unique multi-locus genotypes. Mann-Whitney Rank Sum Test indicated no difference in null allele frequency among loci between the two lineages, N_L1_ = 10, N_L2_ = 10, U = 27.50, T = 82.50, *p* = 0.10). However, there were locus specific differences in null allele frequency for loci BC67 (t = - 3.47, df = 135, *p* < 0.001) and BC36 (t = -2.03, df = 135, *p* = 0.04) when analyzed across all samples.
(EPS)

S5 Fig Principal coordinates analysis of 148 *L*. *glaberrima* genotypes from the GoM. Principal coordinates analysis of the genotypes of *L*. *glaberrima*. To facilitate comparison with Fig 1d the blue (L1) and purple (L2) circles indicate the two microsatellite lineages identified via the STRUCTURE analysis. Shown are the first two axes, explaining 46.05% and 13.27% of the variation, respectively. VK862 excluded (n = 2).
(EPS)

S6 Fig Genotype distances among samples. (a) Minimum Spanning Networks of microsatellite genotype distances among samples (Bruvo distances), calculated for the microsatellite lineages. Clusters of Lineage 1 had a star topology. This pattern is common in recent radiations. Lineage 2 had a branching pattern, concordant with linear descent. The thicker the edges the greater the relatedness between the nodes (based on mutation steps). (b) Neighbor-Joining tree of microsatellite genotype distances among samples (Bruvo distances), calculated for the two microsatellite lineages.
(EPS)

S1 Methods Amplification conditions for mitochondrial and nuclear markers for species delimitation. Amplification conditions for microsatellite markers for population genetics. Spatial analysis. (DOCX)

S1 Table Microsatellite primers for *Leiopathes glaberrima*. Given are: Locus = primer name, Primer sequences with tail (T1, T3) or fluorescent label used, Motif = microsatellite motif, Size (bp) = length of the microsatellite, Plex = some primers were combined in multiplex reactions, Temp = Annealing temperature for the amplification. PCR conditions in S1 Methods.
(DOCX)

S2 Table *Leiopathes glaberrima* microsatellite loci diversity. Given are: N = number of samples genotyped at that locus, N_a_ = No. of Different Alleles, H_o_ = Observed Heterozygosity, H_e_ = Expected Heterozygosity, H_t_ = Total Expected Heterozygosity, F = Fixation Index. F_is_ = (Mean H_e_—Mean H_o_) / Mean H_e_, F_it_ = (H_t_—Mean H_o_) / H_t_, Fst = (H_t_—Mean H_e_) / H_t_. SE = standard error.
(DOCX)

S3 Table Spatial spread of genets (m) for those *L*. *glaberrima* colonies where navigational accuracy was good. N = number of ramets per genet. SD = standard deviation. Navigational accuracy within a single cruise is generally about +/- 5 meters at this depth using USBL alone and about twice that (+/- 10 m) when data from independent visits are combined. In many cases distances between corals was determined using Doppler velocity navigation streams during a single dive and this can result in paired distance estimates accurate to about 0.5 meters.
(DOCX)
