# Supplementary material for: Home Bodies and Wanderers: Sympatric Lineages of the Deep-Sea Black Coral Leiopathes glaberrima
Source: PLoS One. 2015 Oct 21;10(10):e0138989. doi: 10.1371/journal.pone.0138989 (PMC4619277; doi:10.1371/journal.pone.0138989)
Supplement: S1 Methods — (DOCX) [file pone.0138989.s007.docx]

S1 Methods

*Microsatellite design*

Microsatellites (Simple Sequence Repeats) for population connectivity analyses of *L. glaberrima* were designed from sequence data obtained from genomic shotgun sequencing *of L. glaberrima* on a 454 GS-FLX sequencer. Contigs and singletons were imported to the Tandem Repeat Finder (TRF) database and processed using the default values of Match = 2, Mismatch = 7, Indels = 7 as alignment parameters ([39](#_ENREF_39)). Sequences were annotated for perfect motifs using Geneious ([40](#_ENREF_40)); sequences containing both flanking regions of at least 100 bases were imported to Primer 3 for primer design ([41](#_ENREF_41)). Primers were realigned to the sequences with Codon Code Aligner. Candidate loci were tested for polymorphism and reliable amplification on samples from the most geographically distant sites in the GoM (VK 906, GB535 and WFS), resulting in 10 polymorphic loci (Genbank submission numbers KJ914618-KJ914627; S2 Table).

*Amplification conditions for mitochondrial and nuclear markers for species delimitation*

*Leiopathes*-specific mitochondrial markers: COI-COIII, ND5-ND2 and TRP (partial TrnW-ITS-NADH) ([Brugler & France 2007](#_ENREF_10); [Sinniger & Pawlowski 2009](#_ENREF_81)), and coral-specific nuclear ribosomal ITS-1 ([Takabayashi *et al.* 1998](#_ENREF_82)) were used for species identification. For each primer sets, 20 ng of template DNA were added to reaction solutions consisting of 0.2 µM primers (each, IDT/ABI), 1X of Bioline NH4 reaction buffer, 2 mM of MgCl_2_ (Bioline), 0.5 mg/ml of BSA (NEB), 2 mM dNTPs (Bioline) and 1 U Biolase Taq. Polymerase chain reaction (PCR) conditions for COI-COIII included an initial denaturation at 94˚ C for 2 min followed by 30 cycles of denaturation at 94˚ C for 15 sec, annealing at 54˚C for 30 sec and extension at 72˚C for 30 sec, with a 1 min final extension at 72˚C. Conditions for ND5-ND2 were the same but the annealing step was at 55˚C. TRP amplification was performed with 40 cycles of denaturation at 94˚C for 30 sec, annealing at 51˚C for 30 sec and extension at 72˚C for 45 sec with a final extension of 10 min at 72˚C. For the ribosomal ITS-1, the PCR conditions were: initial denaturation at 94˚ C for 2 min followed by 35 cycles of denaturation at 94˚ C for 30 sec, annealing at 52˚C for 30 sec and extension at 72˚C for 45 sec with a final extension of 30 min at 72˚ C.

*Amplification conditions for microsatellite markers for population genetics*

PCR mix for multiplex 1 consisted of 0.05 µM of each primer (BC11, BC22, BC43, BC67, IDT/ABI), 1X NH4 Bioline reaction buffer, 1.5 mM of MgCl_2_ (Bioline), 0.2 mM dNTPs (Bioline) and 1 U Bioline Taq. Multiplex 2 mix was 0.74 µM of primer BC01, 0.1 µM primer BC05, 0.05 µM primer BC08 (IDT/ABI), 1X Bioline NH4 reaction buffer, 1.5 mM of MgCl_2_ (Bioline), 0.2 mM dNTPs (Bioline) and 1 U Bioline Taq. The PCR mix for the singleplexes of primers BC34 and BC36 was 0.05 µM of forward primer, 0.5 µM of reverse primer, 0.25 µM fluorescently labeled primer, 1X Bioline NH4 reaction buffer, 0.75 mM of MgCl_2_, 0.2 mM dNTPs (Bioline) and 0.1 U/µL Bioline Taq. The singleplex for BC19 had the same concentrations of reagents, but 0.05 µM of each primer (IDT/ABI) was used. Between 10 and 20 ng of DNA was added to each reaction. General amplification conditions were: initial denaturation step at 94˚ C for 5 min, followed by 35 cycles of denaturation at 94˚ C for 20 sec, annealing for 20 sec and extension at 72˚C for 30 sec, with a final extension at 72˚C for 30 min. Annealing temperature was 54˚C for multiplex 1 and BC19, 57˚C for multiplex 2, and 55˚C for BC34 and BC36 (Table 1 Supporting Information).

*Spatial analysis*

*Leiopathes* survey transects were derived from frame grabs of down looking video obtained using the ROV Jason II during 2009 and 2010 dives to the VK sites. The frame grabs and location data were obtained from the WHOI Jason VirtualVan, an online repository (http://4dgeo.whoi.edu/jason/) of frame-grabs from the three cameras aboard ROV *Jason* and associated dive data taken at roughly 1-minute intervals. The navigation data is accurate to within about 15 m between lowerings using USBL navigation and within about 1m during a dive based on the acoustic doppler velocity navigation stream. Survey points were manually selected at 10-meter intervals, exported and time-matched to the framegrabs in the VirtualVan. Framegrabs were surveyed for the presence/absence, number and color of *L. glaberrima* colonies. The data was imported into ArcGIS Version 10.1 (ESRI 2011. ArcGIS Desktop: Release 10. Redlands, CA: Environmental Systems Research Institute) and overlaid on bathymetry (VK906 R/V Nancy Foster Multibeam with a 5m pixel resolution, VK826 AUV Sentry Multibeam with a 1m pixel resolution). The site bathymetry was processed through the Spatial Analyst extension to produce slope and aspect raster datasets. This slope dataset was calculated from the rate of z-value change between each raster cell. The aspect (slope orientation) dataset was calculated from the direction of the maximum rate of z-value change from each raster cell. All raster datasets were exported from ArcGIS Version 10.1 into MaxEnt ([Phillips *et al.* 2006](#_ENREF_62)). In addition to the three raster datasets, *L. glaberrima* collections made during the 2009 and 2010 Lophelia II Research Cruises were added to the MaxEnt program. The *L. glaberrima* collections dataset included the color, microsatellite lineage, clone id (as identified via microsatellite genotyping, see below), and the UTM coordinates for each collected colony. Each non-location attribute (color, microsatellite lineage) was modeled with MaxEnt to identify the environmental characteristics associated with the occurrence of *L. glaberrima*. Model training was done using 20% of the non-location data as response variables. Each non-location data set was then run three times with default parameters. The area under the receiver-operated characteristic (AUC) metric ranges from 0 to 1, AUC > 0.9 indicates good model performance ([Fielding & Bell 1997](#_ENREF_27)). When presence-only data is used, AUC values of 0.5 indicate that the discrimination of the model is not better than random. To test whether the model was significantly better then random (AUC > 0.5), a One-tailed One-Sample T-test (or a One-Sample Signed Rank test if normality test failed) was performed.
