# Supplementary material for: Home Bodies and Wanderers: Sympatric Lineages of the Deep-Sea Black Coral Leiopathes glaberrima
Source: PLoS One. 2015 Oct 21;10(10):e0138989. doi: 10.1371/journal.pone.0138989 (PMC4619277; doi:10.1371/journal.pone.0138989)
Supplement: S1 Table — Given are: Locus = primer name, Primer sequences with tail (T1, T3) or fluorescent label used, Motif = microsatellite motif, Size (bp) = length of the microsatellite, Plex = some primers were combined in multiplex reactions, Temp = Annealing temperature for the amplification. PCR conditions in S1 Methods. (DOCX) [file pone.0138989.s008.docx]

**S1 Table** Microsatellite primers for *Leiopathes glaberrima*. Given are: Locus = primer name, Primer sequences with tail (T1, T3) or fluorescent label used, Motif = microsatellite motif, Size (bp) = length of the microsatellite, Plex = some primers were combined in multiplex reactions, Temp = Annealing temperature for the amplification. PCR conditions in S1 Methods.

| Locus | Primer sequence (5'-3') | Motif | Size (bp) | Plex | Temp |
| --- | --- | --- | --- | --- | --- |
| BC1 | F: PET-TAG TAC CCT CGC AGC AGG GTG | (ACT)6 | 182-185 | 2 | 57˚C |
|  | R: GAA TTC CGC TCG TCC TCC A |  |  |  |  |
| BC5 | F: 6FAM-TGA AGA GTG AGC ACT CGT T | (TTG)8.6 | 193-235 | 2 | 57˚C |
|  | R: CAG TAT GTC CGC GTC ATC TT |  |  |  |  |
| BC8 | F: NED-CAC AGT AAG CTG ACC GTC TGC | (TTA)11 | 119-147 | 2 | 57˚C |
|  | R: TAC CGT ATG CCC ACG AAG AGC |  |  |  |  |
| BC11 | F: PET-GAG ACA TGA CTT TCC AGA TCC GCT | (AAC)14 Y (TGTAA)4 | 123-183 | 1 | 54˚C |
|  | R: CGT AAA TCA GCA CAC ATT TCC GGT |  |  |  |  |
| BC22 | F: NED- GCA ACA GAG AGC TTG GTT CAA | (GTT)10 | 142-163 | 1 | 54˚C |
|  | R: CTT CCT GTC CCA ACA CCC T |  |  |  |  |
| BC43 | F: VIC-ATC CTC TGT GGT GTA TGT T | (CCA)5 | 141-147 | 1 | 54˚C |
|  | R: AGT GAT CTC CCA TTC GAC C |  |  |  |  |
| BC67 | F: 6FAM-TCC CTT TCA AGA TCC GTA | (ATT)10 | 133-172 | 1 | 54˚C |
|  | R: CGA CTA CAA TTC GAT CAA CA |  |  |  |  |
| BC19 | F: 6FAM-GCC AAT ATT GCT GCG GTT AC | (GTTGGC)10.5 | 198-309 | single | 54˚C |
|  | R: AAG AGA CAG GTC CGG TTG AA |  |  |  |  |
| BC34 | F: T1-GGC TAG GAA AGG TTA GTG GCC ACA TGT CGT CCT GGA TAC G | (AACAAG)9 | 168-204 | single | 55˚C |
|  | R: GCC TAA CTT TTA CTT TAT TTA TTG CAT |  |  |  |  |
| BC36 | F: T3-ACC AAC CTA GGA AAC ACA GGA GCC CTG AAG GAT CAG AGA | (ACA)4ATAG (TAA)9 | 174-207 | single | 55˚C |
|  | R: AAA AGC ATC TAC GGG TGG TG |  |  |  |  |
