# Supplementary material for: Home Bodies and Wanderers: Sympatric Lineages of the Deep-Sea Black Coral Leiopathes glaberrima
Source: PLoS One. 2015 Oct 21;10(10):e0138989. doi: 10.1371/journal.pone.0138989 (PMC4619277; doi:10.1371/journal.pone.0138989)
Supplement: S2 Table — Given are: N = number of samples genotyped at that locus, Na = No. of Different Alleles, Ho = Observed Heterozygosity, He = Expected Heterozygosity, Ht = Total Expected Heterozygosity, F = Fixation Index. Fis = (Mean He—Mean Ho) / Mean He, Fit = (Ht—Mean Ho) / Ht, Fst = (Ht—Mean He) / Ht. SE = standard error. (DOCX) [file pone.0138989.s009.docx]

**S2 Table** *Leiopathes glaberrima* microsatellite loci diversity. Given are: N = number of samples genotyped at that locus, N_a_ = No. of Different Alleles, H_o_ = Observed Heterozygosity, H_e_ = Expected Heterozygosity, H_t_ = Total Expected Heterozygosity, F = Fixation Index. F_is_ = (Mean H_e_ - Mean H_o_) / Mean H_e_, F_it_ = (H_t_ - Mean H_o_) / H_t_, Fst = (H_t_ - Mean H_e_) / H_t_. SE = standard error.

|  |  | BC1 | BC5 | BC8 | BC11 | BC19 | BC22 | BC34 | BC36 | BC43 | BC67 | Total |
| --- | --- | --- | --- | --- | --- | --- | --- | --- | --- | --- | --- | --- |
| N | Mean | 29.60 | 29.20 | 29.40 | 29.60 | 29.40 | 29.80 | 29.80 | 29.20 | 29.60 | 29.60 | 29.52 |
|  | SE | 9.13 | 8.74 | 8.74 | 9.10 | 8.91 | 9.09 | 9.09 | 8.74 | 8.91 | 9.22 | 2.56 |
| N_a_ | Mean | 2.00 | 6.00 | 5.80 | 11.60 | 12.20 | 4.20 | 5.80 | 4.60 | 2.40 | 8.20 | 6.28 |
|  | SE | 0.00 | 0.71 | 0.58 | 1.33 | 1.07 | 0.73 | 0.80 | 0.93 | 0.24 | 1.24 | 0.53 |
| H_o_ | Mean | 0.29 | 0.76 | 0.49 | 0.87 | 0.90 | 0.26 | 0.57 | 0.56 | 0.30 | 0.69 | 0.57 |
|  | SE | 0.09 | 0.09 | 0.07 | 0.04 | 0.03 | 0.04 | 0.08 | 0.03 | 0.11 | 0.10 | 0.04 |
| H_e_ | Mean | 0.23 | 0.66 | 0.69 | 0.87 | 0.87 | 0.38 | 0.62 | 0.62 | 0.34 | 0.77 | 0.61 |
|  | SE | 0.06 | 0.02 | 0.02 | 0.01 | 0.01 | 0.07 | 0.04 | 0.06 | 0.08 | 0.06 | 0.03 |
| F | Mean | -0.19 | -0.15 | 0.29 | -0.01 | -0.04 | 0.27 | 0.09 | 0.05 | 0.22 | 0.12 | 0.07 |
|  | SE | 0.07 | 0.11 | 0.10 | 0.06 | 0.04 | 0.10 | 0.09 | 0.13 | 0.19 | 0.10 | 0.04 |
| F_is_ |  | -0.26 | -0.16 | 0.29 | 0.00 | -0.04 | 0.32 | 0.08 | 0.10 | 0.10 | 0.10 | 0.05 |
|  | SE |  |  |  |  |  |  |  |  |  |  | 0.06 |
| F_it_ |  | -0.17 | -0.13 | 0.34 | 0.04 | 0.00 | 0.56 | 0.14 | 0.22 | 0.38 | 0.20 | 0.16 |
|  | SE |  |  |  |  |  |  |  |  |  |  | 0.07 |
| F_st_ |  | 0.07 | 0.02 | 0.06 | 0.04 | 0.04 | 0.36 | 0.07 | 0.13 | 0.31 | 0.11 | 0.12 |
|  | SE |  |  |  |  |  |  |  |  |  |  | 0.04 |
