# Supplementary material for: Home Bodies and Wanderers: Sympatric Lineages of the Deep-Sea Black Coral Leiopathes glaberrima
Source: PLoS One. 2015 Oct 21;10(10):e0138989. doi: 10.1371/journal.pone.0138989 (PMC4619277; doi:10.1371/journal.pone.0138989)
Supplement: S3 Table — N = number of ramets per genet. SD = standard deviation. Navigational accuracy within a single cruise is generally about +/- 5 meters at this depth using USBL alone and about twice that (+/- 10 m) when data from independent visits are combined. In many cases distances between corals was determined using Doppler velocity navigation streams during a single dive and this can result in paired distance estimates accurate to about 0.5 meters. (DOCX) [file pone.0138989.s010.docx]

**S3 Table** Spatial spread of genets (m) for those *L. glaberrima* colonies where navigational accuracy was good. N = number of ramets per genet. SD = standard deviation. Navigational accuracy within a single cruise is generally about +/- 5 meters at this depth using USBL alone and about twice that (+/- 10 m) when data from independent visits are combined. In many cases distances between corals was determined using doppler velocity navigation streams during a single dive and this can result in paired distance estimates accurate to about 0.5 meters.

| Site | Clone ID | Mean (m) | SD | Max (m) | N | Msat Lineage |
| --- | --- | --- | --- | --- | --- | --- |
| VK826 | BC1054 | 69 | 79 | 155 | 3 | 2 |
|  | BC1055 | 48 | 46 | 108 | 3 | 2 |
|  | BC1122 | 16 | 19 | 33 | 2 | 2 |
|  | BC1064 | 1 | 1 | 2 | 2 | 2 |
|  | BC1073 | 1 | 1 | 2 | 2 | 2 |
|  | Mean (SD) | 27 (30) |  | 60 (69) |  |  |
| VK906 | BC1093 | 60 | 32 | 131 | 21 | 2 |
|  | BC1094 | 37 | 21 | 75 | 15 | 2 |
|  | BC1095 | 42 | 0 | 42 | 3 | 2 |
|  | BC1096 | 45 | 0 | 45 | 2 | 2 |
|  | BC1097 | 65 | 33 | 107 | 2 | 2 |
|  | BC1098 | 144 | 0 | 144 | 4 | 2 |
|  | BC1110 | 80 | 54 | 118 | 3 | 2 |
|  | BC1117 | 611 | 0 | **611** | 2 | 2 |
|  | Mean (SD) | 135 (195) |  | 159 (186) |  |  |
